# Family Support and Somatic Symptoms: The Serial Mediating Roles of Anxiety and Depression—A Multi-Group Comparison Between Peer Counselors and General Students

**DOI:** 10.3390/bs16091465

**Published:** 2026-08-24

**Authors:** Jin Yu, Haibo Yang, Zhixing Liang

**Affiliations:** 1 Department of Mental Health Guidance Center, Civil Aviation University of China, Tianjin 300300, China; yuj@cauc.edu.cn; 2 Faculty of Psychology, Tianjin Normal University, Tianjin 300387, China; 3 Tianjin Social Science Laboratory of Students’ Mental Development and Learning, Tianjin 300387, China; 4 Academic Affairs Office, Civil Aviation University of China, Tianjin 300300, China; zxliang@cauc.edu.cn

**Keywords:** anxiety, depression, family support, multi-group comparison, somatic symptoms

## Abstract

**Objective:** This study examines the relationship between family support and somatic symptoms and tests whether anxiety and depression serially mediate this relationship differently between peer counselors and general students within an integrated framework of Conservation of Resources Theory, Social Support Theory, and the Gain Spiral mechanism. **Methods:** A total of 2576 peer counselors and 5446 general students completed the University Personality Inventory (UPI), the Depression–Anxiety–Stress Scale (DASS-21), and the Perceived Social Support Scale (PSSS). **Results:** ① Peer counselors reported higher family support and lower depression, anxiety, and somatic symptoms than general students. ② Depression independently mediated the effect in both groups (68.4% vs. 82.6% of the total effect; group difference non-significant). ③ Serial mediation via anxiety and depression was significant and full in general students, but not-significant in peer counselors, where only anxiety acted as an independent mediator. **Conclusions:** The association between family support and somatic symptoms follows distinct pathways across groups. General students exhibit a complete anxiety–depression sequential mediation pathway. In contrast, peer counselors show a simplified mediation pattern alongside a stronger direct association. These findings offer empirical evidence for developing tiered mental health strategies in higher education.

## 1. Introduction

### 1.1. Current Mental Health Status and Somatic Symptoms Among College Students

College students are in a critical period of transition from adolescence to adulthood, facing multiple developmental challenges such as academic pressure, adaptation to interpersonal relationships, emotional distress, and career development (Arnett, 2000). During this vulnerable period, when psychological resilience fluctuates sharply, physical and mental health problems have increasingly attracted public attention. Somatic symptoms refer to experiences of physical discomfort that occur under psychological stress, often manifesting as somatic complaints without a clear organic pathological basis, such as headaches, fatigue, gastrointestinal discomfort, palpitations, and muscle tension. Their essence lies in the fact that when emotional expression is inhibited, psychological stress is ultimately transformed into warning signals at the bodily level through sustained activation of the hypothalamic–pituitary–adrenal (HPA) axis and dysregulation of immune-inflammatory responses (C. Li et al., 2021). The detection rate of somatic symptoms among Chinese college students is far from encouraging. The detection rate of somatic symptoms among Chinese college students exhibits considerable variation across studies, owing to heterogeneity in research instruments, sample characteristics, and assessment criteria. An early survey targeting medical students (using the UPI, *n* = 1069) reported detection rates of mild, moderate, and severe somatic symptoms at 39.17%, 24.14%, and 5.66%, respectively (M. Zhang et al., 2000). By contrast, another survey of 1380 undergraduates at a comprehensive university in Sichuan Province (using the PHQ-15) found that 11.8% of participants reported moderate or above levels of somatic symptoms (Xu et al., 2013). This discrepancy may be partially attributed to differences in sample composition (medical students vs. general undergraduates) and measurement instruments (UPI vs. PHQ-15). In recent years, emotional and behavioral problems among college students have shown a trend toward diversification and increasing complexity (Oswalt et al., 2020). At the international level, a systematic review and meta-analysis incorporating multi-country studies estimated the pooled prevalence of depressive and anxiety symptoms among college students at 33.6% and 39.0%, respectively (W. Li et al., 2022). A national survey focusing specifically on the Chinese college student population (conducted in 2023) reported lower prevalence rates of 9.8% for depressive symptoms and 15.5% for anxiety symptoms (Han et al., 2025). Regional studies have yielded relatively higher figures: the detection rates of anxiety and depression among college students in Jiangxi Province were 43.6% and 63.9%, respectively (Zhou et al., 2024), while those in Lanzhou City were 48.3% and 22.9%, respectively (Y. Yang et al., 2024). These regional discrepancies are likely associated with differences in the scales and cut-off values adopted across studies, and also suggest that geographic factors may exert a non-negligible influence on the assessment of college students’ mental health status.

Although mental health problems among college students have become increasingly prominent, the utilization rate of professional psychological services remains persistently low. According to the Report on National Mental Health Development in China (2023–2024) (Fang et al., 2025), which collected 60,782 valid responses nationwide, 80.0% of college students were aware that their institutions provided free psychological counseling, yet the actual utilization rate was only 2.8%. The report also indicated that emotional and instrumental support from roommates significantly reduced the risks of anxiety and depression, suggesting that peer support constitutes an important psychological protective resource. Against this background, support systems that do not rely on individuals’ active help-seeking initiative yet function continuously in daily life merit particular attention. Family support, as the most fundamental and stable component of an individual’s social support system, operates independently of formal help-seeking channels and has been extensively validated by empirical research to facilitate psychological adaptation (X. Liu & Huang, 2010; D. Chen et al., 2026). Accordingly, the present study focuses on family support as a non-formal resource, examining its pathways to somatic symptoms through the independent and serial mediating roles of anxiety and depression, and comparing these pathways between peer counselors and general students through multi-group analysis, with the aim of providing empirical evidence for the development of tiered mental health intervention systems in higher education contexts where professional help-seeking is constrained.

### 1.2. Protective Mechanisms of Family Support

Family support is the most fundamental and stable component of an individual’s social support system, and its protective function has received support from multiple theoretical perspectives. Attachment theory suggests that family support provides a secure emotional attachment foundation, enabling individuals to perceive understanding and acceptance in social interactions, thereby buffering anxiety (Y. Liu et al., 2025). Social support theory further distinguishes between enacted support and perceived support. The emotional warmth, tangible assistance, and informational guidance provided by family can not only directly enhance mental health through the main effect model but also mitigate the adverse impact of negative emotions under stressful conditions through the buffering effect model (X. Liu & Huang, 2010). A substantial body of empirical research has demonstrated that perceived support exhibits stronger and more stable predictive power for mental health outcomes than enacted support, as it more directly shapes individuals’ cognitive appraisals and emotional experiences (X. Liu & Huang, 2010; X. Wang et al., 2026). Accordingly, the present study operationalizes family support as perceived family support, that is, individuals’ subjective evaluation of the availability of emotional care, tangible assistance, and informational guidance from family members.

A substantial body of empirical evidence has consistently demonstrated the stable protective effect of family support on college students’ mental health. Cumulative family risk positively predicted internalizing psychological problems among college students, with perceived social support serving as a mediator (Lu et al., 2025); intolerance of uncertainty was positively associated with anxiety levels, a relationship that family support effectively buffered (X. Chen & Chen, 2025); early-life adversity positively predicted anxiety and somatic symptoms, and family support attenuated its long-term negative effects (X. Wang et al., 2026); social support negatively predicted anxiety and depression, with perceived loss of control playing a partial mediating role (Shi, 2024); family intimacy was negatively associated with negative emotions, with social support and psychological resilience constituting a chain mediation pathway (D. Chen et al., 2026).

However, the above studies all focused on general college student populations, conceptualizing family support as a static external resource and assuming that its protective mechanisms follow a homogeneous pathway across individuals. This perspective fails to address a more theoretically nuanced question: Does the protective effect of family support vary in its conversion efficiency as a function of differences in individual psychological resources? In particular, when individuals have a specialized background in psychological training, does this conversion process exhibit differences in pathway or advantages in efficiency? This theoretical blind spot leaves room for subsequent comparative studies across groups.

### 1.3. Psychological Advantages of Peer Counselors

Most current studies have focused exclusively on samples of general college students, with relatively limited attention to the special group of peer counselors. Peer counselors in higher education institutions are composed primarily of psychology class representatives, dormitory leaders, and class and Youth League student leaders. After receiving systematic psychological training, they are embedded in students’ everyday lives and constitute a sentinel force for early warning of psychological crises. A small number of empirical studies have indicated the mental health advantages of this group: the physical and mental health levels of class psychology representatives are higher than those of ordinary student leaders (J. Zhang, 2015); a randomized controlled trial by Y. Chen et al. (2017) demonstrated that peer support training can reduce depression and anxiety and enhance perceived social support; the competence of psychology class representatives can influence mental health through the chain mediating effects of perceived social support and resilience (J. Wang et al., 2021); and parent–child attachment positively predicts the competence of psychology class representatives, with interpersonal acceptance playing a mediating role, suggesting the existence of a resource transformation pathway between family support and peer helping capacity (Yu et al., 2023).

Although the studies reviewed above have confirmed the advantages of peer groups in mental health indicators, three key issues remain unresolved. First, whether there exist structural differences in pathway patterns—that is, differences manifested in the presence versus absence or relative strength of pathways, rather than merely in the magnitude of effect sizes—in the mediating pathways through which family support influences somatic symptoms across groups has yet to be directly addressed by multigroup chain mediation comparative analyses. Second, whether anxiety and depression play temporally distinct roles in this process (anxiety as an immediate stress response, depression as a long-term cumulative emotional state), and whether the conversion patterns of these two emotional pathways differ between the two groups, require further theoretical elaboration and empirical examination. Third, whether the anxiety–depression cascade pathway exhibits a pattern in peer groups that differs from that in general students needs to be verified through statistical group coefficient comparisons, rather than relying solely on descriptive comparisons of significance status across individual groups. These three issues collectively constitute the core theoretical gap addressed by the present study, which will conduct formal statistical inferences regarding group differences in the above pathways through multigroup mediation comparison.

### 1.4. Theoretical Integration Framework

This study integrates three theoretical perspectives into an analytical framework, with each theory assuming a distinct functional role in explaining specific pathways.

Conservation of Resources Theory is the overarching framework. Conservation of Resources (COR) Theory posits that individuals consistently strive to acquire, maintain, and protect the resources they value, and that resource loss or insufficiency leads to psychological stress and psychosomatic symptoms (Hobfoll, 1989). This theory provides the overarching explanatory logic for the present study: perceived family support, as a critical evaluation of external resources, is associated with individuals’ psychological adaptation status; when individuals perceive sufficient resources, psychological distress levels are low, whereas perceived resource scarcity or the threat of resource loss is accompanied by elevated anxiety, depression, and somatic symptoms. Accordingly, COR Theory establishes the fundamental logical premise of this study: differences in individuals’ perceived family support are associated with their emotional states and somatic symptom levels.

Social Support Theory is the explainer of mediating pathways. Social Support Theory further clarifies the pathways through which perceived family support is associated with mental health. This theory posits that social support influences mental health through two pathways: the main effect model, whereby social support is directly associated with mental health levels irrespective of the presence of stressful conditions; and the buffering effect model, whereby social support interacts with the detrimental effects of negative emotions on mental health under stressful conditions (X. Liu & Huang, 2010). In the present study, these two pathways correspond respectively to: a direct association between perceived family support and somatic symptoms and an indirect association between perceived family support and somatic symptoms through the alleviation of anxiety and depression. Thus, Social Support Theory provides the theoretical grounding for the specific mediating pathways examined in this study.

The Gain Spiral mechanism as the explainer of group differences. The Gain Spiral mechanism, as a dynamic extension of COR Theory, is specifically employed to explain the pathway differences between peer counselors and general students in this study. This mechanism posits that, in the process of resource investment, existing resources can generate new resources, forming a positive cycle of resource gain, further investment, and more resources (Hakanen et al., 2008). Based on this reasoning, peer groups, through systematic psychological training and helping practices, exhibit positive associations between perceived family support and internal psychological capital, thereby forming an accelerated gain spiral. In contrast, general groups, lacking this specialized transformation pathway, exhibit a resource transformation pattern more characterized by a single emotional relief pathway (Halbesleben & Wheeler, 2015; H. Wang et al., 2022; Jia et al., 2024). The Gain Spiral mechanism thus provides the theoretical rationale for predicting group differences: peer groups should exhibit a stronger direct effect and different mediation patterns in the association between perceived family support and somatic symptoms.

In summary, the three theories play distinct roles within the analytical framework: COR Theory provides the overall logical premise, Social Support Theory explains the specific emotional mediating pathways, and the Gain Spiral mechanism explains group differences between peer counselors and general students. This layered integration framework lays the theoretical foundation for subsequent hypothesis formulation and multigroup comparison analyses.

### 1.5. The Sequential Cascade of Anxiety and Depression: A Chain-Mediated Model

Anxiety and depression are highly co-occurring, progressively developing negative emotions, yet their temporal ordering remains empirically debated. According to a longitudinal study with four time points over three years by Long et al. (2018), physical anxiety symptoms and depressive symptoms reciprocally predicted each other, and social anxiety and depression symptoms similarly exhibited a systematic reciprocal pattern, suggesting that the development of these syndromes occurs in a predictable, systematic reciprocal manner rather than sequentially and unidirectionally. Meta-analytic evidence (Jacobson & Newman, 2017) also indicates a bidirectional risk relationship between anxiety and depression. While some studies have found that anxiety serves as a sequential mediator linking stress to depression in non-clinical adult populations, these findings are derived from specific contextual samples (Marcos-Escobar et al., 2025). Given the mixed evidence regarding temporal precedence, the present study adopts a theoretical rather than causal perspective on the anxiety–depression relationship: anxiety is conceptualized as more closely associated with immediate stress arousal, whereas depression corresponds to the cumulative psychological toll over time (Tang & Kuang, 2009). This theoretical distinction provides the rationale for positioning anxiety as the earlier link in the emotional cascade examined in our mediation model.

The chain model of social support → anxiety → depression → suicidal ideation verified by H. Chen et al. (2025) provides methodological support for including anxiety and depression simultaneously into the mediation framework. X. Chen and Chen (2025) further pointed out that peer supporters who have received psychological training exhibit stronger abilities to identify and regulate their own and others’ anxiety; the bidirectional helping others and self-help interaction model may enable them to superimpose additional psychological resources when benefiting from family support, particularly at the key node of the anxiety–depression pathway (X. Chen & Chen, 2025). This implies a theoretically meaningful hypothesis: general students may highly rely on the complete emotional cascade path of anxiety–depression to ultimately manifest somatic symptoms, whereas peer counselors may achieve emotional blocking at the anxiety link, which weakens or even eliminates the subsequent depression transformation pathway.

Anxiety and depression are highly coexisting progressive negative emotions. Anxiety often precedes depression, which corresponds to immediate stress arousal; depression corresponds to long-term cumulative psychological trauma, which has a clear time hierarchy (Tang & Kuang, 2009). From the perspective of time series, anxiety is more closely related to current stress, while depression is related to long-term cumulative adverse experiences, which directly supports the positioning of anxiety as an earlier link in the emotional cascade (Jiang et al., 2026). The chain model of social support, anxiety, depression, and suicidal ideation verified by H. Chen et al. (2025) provides methodological support for this study to include anxiety and depression into the mediation framework at the same time.

X. Chen and Chen (2025) further pointed out that peer supporters who have received psychological training have stronger ability to identify and adjust their own and others’ anxiety. The two-way interaction mode of helping others and self-help may enable them to superimpose additional psychological resources when they benefit from family support, especially at the key node of the transformation from anxiety to depression (X. Chen & Chen, 2025). This implies a hypothesis with theoretical depth: the ordinary group may highly rely on the complete emotional cascade path of anxiety–depression to finally show somatic symptoms, while the peer group may achieve emotional blocking in the anxiety link, which weakens or even eliminates the subsequent depression transformation path.

Based on the above literature review and theoretical integration, this study proposes the following assumptions:

**H1.**  *There are significant differences in family support, anxiety, depression, and somatic symptoms between the peer counselors and the general students. Specifically, peer counselors have higher family support and lower anxiety, depression, and somatic symptoms.*

**H2.**  *Depression plays an independent mediating role between family support and somatic symptoms, and this mediating effect is significant in both peer counselors and general students.*

**H3.**  *Anxiety and depression constitute a sequential mediation pathway between perceived family support and somatic symptoms, and the indirect effect of this chain mediation differs significantly between peer counselors and general students. Specifically, the chain mediation effect is significant in the general students, whereas it is significantly weaker in the peer counselors.*

## 2. Methods

### 2.1. Participants

Using convenience sampling, undergraduate students enrolled at a university in Tianjin were recruited as participants and assessed via an online platform (Wenjuanxing, www.wjx.cn) from March to May 2025. The participating university is a comprehensive institution with disciplines spanning engineering, science, humanities, management, and law, characterized by a diverse student body. The university maintains a mental health education center that provides a standardized training system for peer counselors.

Recruitment was conducted through multiple channels: (1) formal notices distributed by the mental health education center via class-based peer counselor workgroups; (2) mental health education instructors promoted the survey during classroom sessions; and (3) peer counselor leaders disseminated the questionnaire link through their respective peer support networks. All participants were informed of the study’s purpose and assured of the confidentiality of their responses prior to providing informed consent. To prevent duplicate responses, each IP address was restricted to a single submission.

This study utilized questionnaire data that underwent anonymization processing and did not involve sensitive information. It did not involve any human intervention, did not collect any sensitive personal privacy information, and had no commercial interest associations. The study fully complied with Article 32 of the Measures for Ethical Review of Life Sciences and Medical Research Involving Human Beings (jointly issued by China’s National Health Commission, the Ministry of Education, the Ministry of Science and Technology, and the State Administration of Traditional Chinese Medicine on 18 February 2023). During the data collection process, this study fully respected participants’ right to autonomy. Participants could withdraw unconditionally at any stage of the survey, and their data would not be recorded or saved. No item-level missing data were present in the final valid sample included for analysis. All participants provided informed consent after being fully informed of the study content.

The peer counselors in this study refer to students serving as class psychological commissioners, dormitory psychological liaisons, or peer psychological counselors at the university level. All of the above members had completed the standardized 8 h psychological training uniformly organized by the university mental health guidance center. The training was delivered by qualified instructors with standardized teaching credentials, covering the identification of common psychological problems among college students, listening and empathy skills, psychological crisis prevention and intervention, as well as strategies for coping with common psychological distress. A rigorous assessment system was implemented: participants were required to pass a theoretical knowledge written examination with a minimum passing score of 85 out of 100 to obtain the training certificate and be authorized to serve as peer counselors. In the Chinese higher education context, these roles are collectively referred to as peer counselors, as they share the core functions of frontline crisis identification, peer emotional support provision, and referral facilitation under professional supervision. This operationalization is consistent with prior research on college peer psychological leaders in China (J. Wang et al., 2021; Yu et al., 2023). All peer counselors included in the present study met the full attendance requirement and passed the unified competency assessment, ensuring that they possessed a basically consistent level of peer psychological helping competence. The general students referred to students who had not participated in any university- or class-level peer psychological training and had no work experience related to psychological services.

A total of 10,945 questionnaires were distributed. Among them, 3545 were distributed to the peer group; after excluding 969 invalid questionnaires, 2576 valid questionnaires were recovered, yielding a valid response rate of 72.7%. A total of 7400 were distributed to the general student group; after excluding 1954 invalid questionnaires, 5446 valid questionnaires were recovered, yielding a valid response rate of 73.6%. The overall valid response rate for the questionnaires was 73.3%. Invalid responses with excessively short completion times or patterned responding were excluded. Demographic information is presented in Table 1.

### 2.2. Measures

#### 2.2.1. University Personality Inventory

The University Personality Inventory (UPI), developed by the Japanese National Conference on Student Counseling, is a mental health screening instrument designed for the early detection of psychological problems among college students. The inventory consists of 60 items scored on a dichotomous scale (“yes” = 1, “no” = 0). The somatic symptoms subscale was used to assess participants’ physical discomfort experiences, comprising 16 items (item numbers: 1, 2, 3, 4, 16, 17, 18, 19, 31, 32, 33, 34, 46, 47, 48, 49), covering somatic complaints such as headaches, neck and shoulder soreness, chest tightness and pain, palpitations, gastrointestinal discomfort, insomnia, fatigue, cold sweats, and dizziness (Takahashi et al., 1995). Total scores range from 0 to 16, with higher scores indicating more severe somatic symptoms. The UPI demonstrates good internal consistency reliability (Nakai et al., 2007). In this study, the Cronbach’s alpha coefficient for the somatic symptoms subscale was 0.80 in both groups.

#### 2.2.2. Depression Anxiety and Stress Scale

The Depression Anxiety and Stress Scale (DASS-21), developed by Lovibond (P. F. Lovibond & Lovibond, 1995) and Lovibond (S. Lovibond & Lovibond, 1995), is the short form of the original 42-item version. The present study employed the Chinese version revised by Gong et al. (2010). The scale consists of three subscales (depression, anxiety, and stress), each containing 7 items. The anxiety subscale assesses autonomic arousal, panic responses, and subjective anxiety experiences, while the depression subscale assesses dysphoria, hopelessness, anhedonia, and negative self-concept. Items are rated on a 4-point Likert scale (0 = did not apply to me at all, 3 = applied to me very much or most of the time) based on the past week. Subscale scores were calculated by summing the responses to the relevant items (raw score range: 0 to 21). Higher scores indicate more intense experiences of the corresponding emotional dimension. The Chinese version of the DASS-21 has demonstrated sound psychometric properties in Chinese college student samples, with the three-factor structure confirming the theoretical framework (Gong et al., 2010). In this study, the Cronbach’s alpha coefficients for the anxiety subscale were 0.87 and 0.88 in the peer group and the general student group, respectively, and the Cronbach’s alpha coefficients for the depression subscale were 0.89 in both groups.

#### 2.2.3. Perceived Social Support Scale

The Perceived Social Support Scale (PSSS) was developed by Zimet et al. (1988), and the present study employed the Chinese version revised by X. J. Yang et al. (2004). The scale consists of 12 items, divided into three dimensions: family support, friend support, and significant other support, with 4 items in each dimension. The family support subscale includes the following four items: “My family really tries to help me” (Item 3), “My family is willing to help me make various decisions” (Item 4), “I can get the emotional help and support I need from my family” (Item 8), and “My family is willing to discuss my problems with me” (Item 11). Items are rated on a 7-point Likert scale (1 = very strongly disagree, 7 = very strongly agree), and participants were instructed to rate each item according to their actual circumstances. The total score for the family support subscale ranges from 4 to 28, with higher scores indicating higher levels of perceived family support. The Chinese version of the PSSS has demonstrated good reliability and validity in Chinese populations, with a Cronbach’s α coefficient of 0.84 for the total scale and subscale coefficients ranging from 0.81 to 0.82 (F. Zhang et al., 2018). In this study, the Cronbach’s α coefficients for the family support subscale were 0.91 and 0.92 in the peer group and the general student group, respectively.

### 2.3. Statistical Analysis

SPSS 27.0 was used for descriptive statistics and group difference tests, and Mplus 7.0 was used for multigroup mediation analysis. All variables in the model (family support, anxiety, depression, and somatic symptoms) were treated as continuous observed variables, with somatic symptoms operationalized as the total score of the UPI somatization subscale (range: 0–16). Parameters were estimated using maximum likelihood (ML) estimation, and standardized coefficients were reported. All analyses controlled for gender and grade. Gender and grade level were controlled for in the analyses, coded as 0 = male and 1 = female for gender, and 1 = freshman through 4 = senior for grade. Because the mediation model was a saturated model (with zero degrees of freedom), traditional fit indices were not applicable. Accordingly, the significance of indirect effects was tested using bias-corrected bootstrap 95% confidence intervals based on 5000 resamples (Hayes, 2018). All indirect effects were based on observed variables, without involving latent variable models. The detailed Mplus command syntax has been provided as Supplementary Materials to ensure the replicability of the analyses (See Supplementary Materials).

## 3. Results

### 3.1. Control and Testing of Common Method Bias

Because the data in this study were collected through self-report measures, the possibility of common method bias exists. Harman’s single-factor test was conducted separately for the peer group and the general student group to examine common method bias. The results showed that the variance explained by the first factor was 28.3% and 28.0%, respectively, both below the 40% threshold, indicating that this study did not have a substantial common method bias problem. In addition, procedural remedies were implemented during data collection, including anonymous responses, reverse-scoring of selected items, and dispersed arrangement of different scales. Taken together, the Harman test results and these procedural remedies suggest that common method bias does not pose a serious threat to the main conclusions of this study.

### 3.2. Multicollinearity Test

To examine multicollinearity among the predictor variables, this study conducted collinearity diagnostics separately for the data from the two groups. The variance inflation factors (VIFs) for the predictor variables in the peer group and the general student group ranged from 1.12 to 4.08 and from 1.10 to 3.97, respectively, all well below the critical value of 5 (O’Brien, 2007). Therefore, this study did not have a serious multicollinearity problem, and the predictor variables could be simultaneously included in the model analysis.

### 3.3. Difference and Correlation Analyses

The results of the independent-samples t test indicated that family support was significantly higher in the peer group than in the general student group, whereas depression, anxiety, and somatic symptoms were significantly lower than in the general student group (see Table 2).

The results of the Pearson correlation analysis showed significant negative correlations between family support and depression, anxiety, and somatic symptoms in both groups, and significant positive pairwise correlations among anxiety, depression, and somatic symptoms (see Table 3).

### 3.4. Multiple-Group Mediation Analysis

After controlling for gender and grade, the mediation effects were tested separately for the two groups using the bootstrap sampling method with 5000 resamples.

#### 3.4.1. Group Comparison of the Mediating Effect of Depression

**Figure 1:**
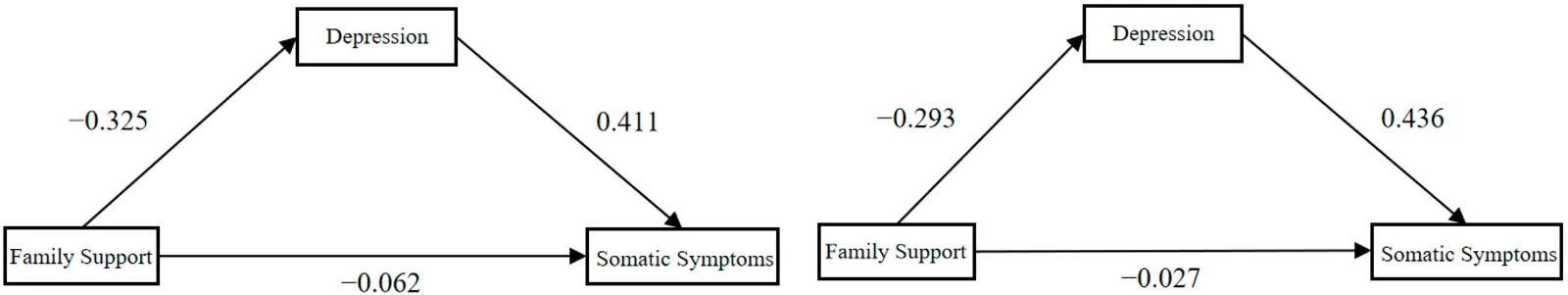
Mediating effect of depression on family support and somatic symptoms (**left**: peer counselor; **right**: general students). Note: All path coefficients shown are standardized.

First, the model with depression as a single mediator was tested, as shown in Table 4 and Figure 1. The results indicated that the mediating effect of depression was significant in both groups, and that both effects were partial mediations. In the peer group, the direct effect of family support on somatic symptoms was −0.062, *p* < 0.01, 95% CI [−0.104, −0.021], accounting for 31.6% of the effect; the indirect effect was −0.134, *p* < 0.001, accounting for 68.4% of the effect. In the general student group, the direct effect was −0.027, *p* < 0.05, 95% CI [−0.052, −0.002], accounting for 17.4% of the effect; the indirect effect was −0.128, *p* < 0.001, accounting for 82.6% of the effect. Multigroup comparisons showed that the differences between the two groups in the direct effect (difference = −0.036, SE = 0.025, *p* = 0.144, 95% CI [−0.083, 0.011]), indirect effect (difference = −0.006, SE = 0.016, *p* = 0.713, 95% CI [−0.038, 0.024]), and total effect (difference = −0.042, SE = 0.027, *p* = 0.123, 95% CI [−0.095, 0.011]) did not reach statistical significance.

#### 3.4.2. Group Comparison of the Serial Mediation Effect of Anxiety and Depression

**Figure 2:**
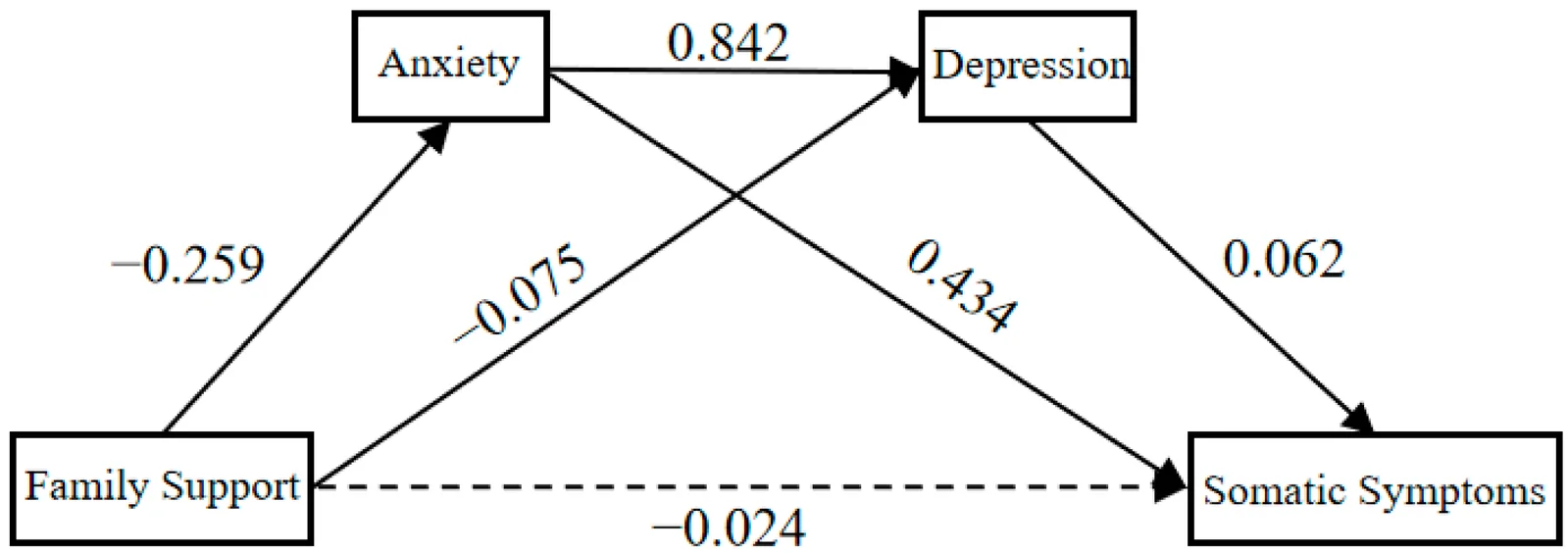
The serial mediation effect of anxiety and depression on family support and somatic symptoms among the general students. Note: All path coefficients shown are standardized.

After anxiety and depression were simultaneously included in the serial mediation model, the mediation pattern changed substantially, with the two groups showing distinctly different patterns, as shown in Table 5 and Figure 2.

For the peer counselors, family support also significantly and negatively predicted depression (β = −0.300, *p* < 0.001). When family support and depression were simultaneously entered to predict anxiety, both factors exhibited significant predictive effects (β = −0.071, *p* < 0.001 for family support; β = 0.845, *p* < 0.001 for depression). However, when family support, depression, and anxiety were all included in the regression equation to predict somatic symptoms, the results showed that only depression had a significant positive predictive effect (β = 0.572, *p* < 0.001), whereas the predictive effects of family support (β = −0.051, *p* < 0.05) and anxiety (β = −0.081, *p* = 0.060) were not significant. The models explained 9.3% of the variance in depression (R^2^ = 0.093, F = 87.94, *p* < 0.001), 75.8% of the variance in anxiety (R^2^ = 0.758, F = 2013.81, *p* < 0.001), and 28.3% of the variance in somatic symptoms (R^2^ = 0.283, F = 203.13, *p* < 0.001) for the peer counselor group.

For the general students, family support significantly and negatively predicted depression (β = −0.259, *p* < 0.001). When family support and depression were simultaneously entered to predict anxiety, both factors exhibited significant predictive effects (β = −0.075, *p* < 0.001 for family support; β = 0.842, *p* < 0.001 for depression). Subsequently, when family support, depression, and anxiety were all included in the regression equation to predict somatic symptoms, the results showed that depression (β = 0.434, *p* < 0.001) and anxiety (β = 0.062, *p* < 0.05) each had a significant positive predictive effect on somatic symptoms, whereas the direct effect of family support on somatic symptoms was not significant (β = −0.024, *p* = 0.054).

**Figure 3:**
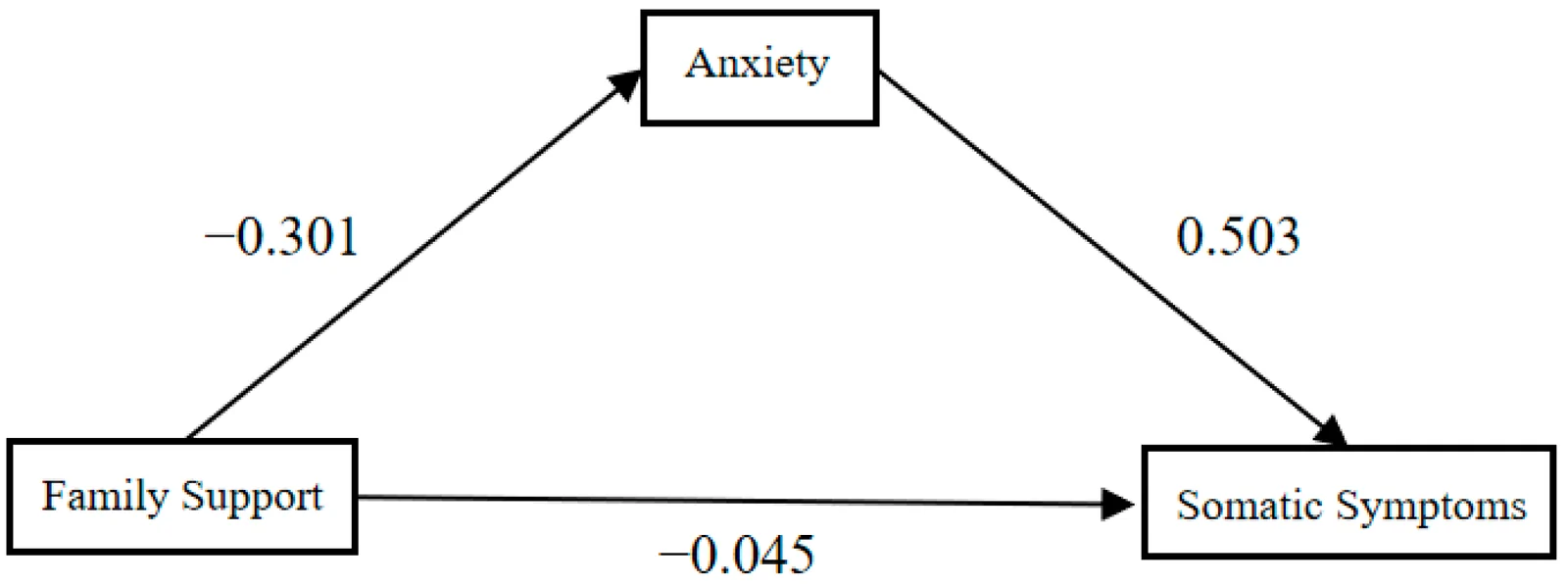
Simple mediating effect of anxiety on the relationship between family support and somatic symptoms in peer counselors. Note: All path coefficients shown are standardized.

Based on the bias-corrected percentile Bootstrap procedure (with 5000 resamples) employed to test the mediation effects (see Table 6), the results indicated that in the peer counselor group, the indirect effect via anxiety was significant (β = −0.172, 95% CI [−0.211, −0.139]). However, the indirect effect via depression was not significant (β = 0.006, 95% CI [−0.0004, 0.013]), nor was the serial mediation effect via both anxiety and depression (β = 0.021, 95% CI [−0.001, 0.043]). The direct effect of family support on somatic symptoms was significant (β = −0.051, 95% CI [−0.093, −0.012]). These results indicated that the mediating effects of anxiety and depression were not significant in the peer counselors. Therefore, anxiety was tested as an independent mediator in the peer counselors (see Table 7 and Figure 3). The direct effect was −0.045 (*p* < 0.05), with a 95% confidence interval of [−0.048, 0.000], which also contained zero, suggesting that anxiety played an independent mediating role in the peer counselors.

In the general students, the indirect effect consisted of three pathways: via anxiety (β = −0.112, 95% CI [−0.133, −0.094]), via depression (β = −0.005, 95% CI [−0.009, −0.0003]), and via the serial mediation of anxiety and depression (β = −0.013, 95% CI [−0.026, −0.001]). Furthermore, the direct effect of family support on somatic symptoms was not significant (β = −0.024, 95% CI [−0.048, 0.000]), indicating that anxiety and depression played a full mediating role in the general students.

The results of the multigroup mediation difference analysis indicated that the between-group differences in all three indirect paths reached statistical significance (see Table 8). The family support → anxiety → somatic symptoms path was significantly smaller (more negative) in the peer counselors than in the general students (difference = −0.059, 95% CI [−0.102, −0.020]); the family support → depression → somatic symptoms path was significantly larger in the peer counselors than in the general students (difference = 0.010, 95% CI [0.003, 0.019]); and the family support → anxiety → depression → somatic symptoms chain path was significantly larger in the peer counselors than in the general students (difference = 0.034, 95% CI [0.010, 0.060]). But the between-group differences found in the direct effect and total effect were not significant.

## 4. Discussion

### 4.1. Group Differences: Resource Transformation Advantages of Peer Counselors

Family support had a significant protective effect against somatic symptoms in both groups. Moreover, the peer group reported significantly higher levels of family support than the general student group, as well as significantly lower levels of depression, anxiety, and somatic symptoms, thereby supporting H1. This group difference has received multifaceted empirical support in previous research. At the level of direct comparison, J. Zhang (2015) found that class psychological monitors exhibited better physical and mental health than other student leaders. At the level of experimental manipulation, the cluster-randomized controlled trial conducted by Y. Chen et al. (2017) showed that participation in peer psychological mutual assistance significantly reduced depression and anxiety among college students and enhanced social support. At the level of mechanism analysis, J. Wang et al. (2021) revealed that psychological monitors’ competence could influence mental health through the chain mediating effects of perceived social support and psychological resilience. Longitudinal research has also confirmed that friend support has moderate effects on the association between socioeconomic status and depression (Cheng & Yang, 2024). Taken together, this evidence suggests a stable association between peer leader status and more favorable psychosocial resources, as well as lower emotional distress.

The “gain spiral” mechanism in conservation of resources theory provides a strong explanatory framework (Hakanen et al., 2008). Compared with general students, peer counselors have received systematic psychological training and have accumulated experience in helping practices that involve emotion regulation and cognitive restructuring. These characteristics are associated with greater psychological capital. Furthermore, peer counselors appear to be more capable of transforming family support into internal resources such as resilience and self-efficacy, thereby facilitating a positive gain spiral. The study by Yu et al. (2023) corroborates this logic, finding that parent–child attachment significantly and positively predicted the competence of psychological committee members, with interpersonal acceptance serving as a mediator. This finding suggests a link between the quality of family support and peer helping capacity. After controlling for mediators such as depression and anxiety, the direct effect of family support on somatic symptoms remained significant in the peer counselors, suggesting that family support is associated with psychological adjustment in peer counselors above and beyond its association with emotional distress.

By contrast, because ordinary students lack systematic psychological training and helping practice, their pattern of resource use may differ from that of peer counselors, and they appear to rely more heavily on a single emotional pathway: family support → alleviation of negative emotions → improvement in somatic symptoms. Once the statistical model accounts for mediators such as depression and anxiety, the independent contribution of family support is no longer significant. The findings of X. Chen and Chen (2025) indirectly support this view: family support is associated with lower anxiety, whereas friend support may sometimes be associated with higher anxiety levels. This suggests that the protective role of family support is distinctive, but its full association with positive outcomes may depend on individuals’ psychological resources: an area in which peer counselors demonstrate an advantage.

Admittedly, the peer counselors in this study were formed through voluntary registration or selection rather than random assignment. Therefore, there may be unmeasured baseline differences between the two groups in terms of mental health, personality traits, motivation, and perceived family support, which could partly account for the observed effects. The psychological resource advantages and emotional transformation patterns exhibited by the peer group may be associated with their training and helping practice experiences, but they may also partly reflect their pre-existing characteristics or selection effects.

### 4.2. The Independent Mediating Effect of Depression

The mediating effect of depression was significant in both groups, with effect proportions of 68.4% and 82.6%, respectively. But the group difference did not reach statistical significance (*p* = 0.713), indicating that the mediating effect of depression demonstrates cross-group consistency between the two groups, thereby supporting H2.

In the general students, the direct effect of family support on somatic symptoms remained significant after depression was entered into the model, but the effect size was substantially reduced relative to the total effect, suggesting that the association between family support and somatic symptoms is largely related to depressive emotions. This pattern is consistent with prior studies on general college students, indicating that depression serves as a key internalizing pathway linking environmental resources to individual psychological adjustment (Pan et al., 2025). For example, Tan et al. (2024) found, in a sample of 1182 college students, that parental autonomy support was associated with lower levels of depression, and this association was mediated by mindfulness and self-esteem. Similarly, Qi et al. (2024), based on a study of 1862 college students, reported that depressive symptoms mediated the association between family functioning and social media addiction. Shi (2024) further showed that social support was negatively associated with anxiety and depression, with perceived loss of control playing a partial mediating role. Taken together, this evidence suggests that for general college students who have not received systematic psychological training, family support is primarily associated with mental health outcomes through its relationship with depressive symptoms—which appear to be a relatively central form of emotional distress in this population (X. Liu & Huang, 2010). In other words, depressive mood appears to be an important pathway through which family support is linked to mental health outcomes; when this pathway is statistically accounted for, the unique association between family support and somatic symptoms becomes considerably weaker.

In the peer counselors, however, depression played a partial mediating role, and the direct effect was relatively larger. One possible explanation for this difference is as follows. First, through systematic training in mental health knowledge and helping skills, peer group members, over the course of long-term peer support practice, may gradually develop more mature emotional cognition and regulation strategies. J. Wang et al. (2021) found that the competence of psychological committee members was significantly positively correlated with perceived social support and psychological resilience, suggesting that individuals with higher competence may have stronger capacities to integrate psychological resources when encountering external support. This implies that when receiving family support, peer counselors may not only regard it as emotional comfort but also more actively apply the psychological regulation skills they have learned, thereby partially bypassing the full mediation of depressive emotions and maintaining a more direct positive association with mental health outcomes. Second, the gain spiral mechanism within the conservation of resources theory (Hakanen et al., 2008) offers a complementary theoretical perspective on this difference. The psychological resources and self-efficacy accumulated by peer counselors through helping practices may facilitate the integration of external family support with internal psychological capital, thereby forming a positive cycle between resource investment and resource acquisition. This dynamic mechanism may allow family support not only to operate through the indirect pathway of alleviating depression but also to maintain a more direct positive association with the psychological adaptation system, thus exhibiting a stronger direct effect. F. Yang et al. (2025) also found that resilience mediated the association between physical exercise and depression, while family support moderated this mediating effect, suggesting that the interaction between family support and individuals’ internal psychological capital may help buffer the risk of depression. For peer counselors, psychological training experience may confer greater advantages in integrating psychological resources across these multiple pathways.

### 4.3. The Mediating Path Between Anxiety and Depression: Independent Mediating Advantage and Emotional Cascade Dependence

After anxiety and depression were simultaneously incorporated into the chain mediation model, the two groups exhibited markedly different mediation patterns. Among general students, the direct effect of family support on somatic symptoms was not significant, whereas the chain mediation pathway of “family support → anxiety → depression → somatic symptoms” was significant and indicated full mediation. This finding suggests that family support must exert its protective effect through a sequential emotion pathway in which anxiety is first alleviated and depression is subsequently reduced, thereby supporting H3.

This sequential pathway is consistent with the theoretical distinction between anxiety and depression in terms of temporal hierarchy: anxiety is conceptualized as a state more closely associated with immediate stress arousal, whereas depression corresponds to the cumulative psychological burden over time (Tang & Kuang, 2009). The chain model of “social support → anxiety → depression → suicidal ideation” verified by H. Chen et al. (2025) provides methodological support for including anxiety and depression simultaneously into the mediation framework. A sequential mediation analysis based on 9796 college students similarly revealed that anxiety is more closely associated with current stress, while depression is related to long-term cumulative adverse experiences—a distinction that, at the theoretical level, supports positioning anxiety as the relatively earlier link in the emotional cascade (Jiang et al., 2026; Tan et al., 2024). It should be noted, however, that these findings are based on cross-sectional data, and the observed “anxiety → depression” sequence reflects statistical associations among variables rather than temporal precedence. For general students who lack systematic psychological knowledge and emotion regulation skills, the protective effect of family support may depend heavily on this complete emotional cascade pathway—anxiety alleviation may be a key starting point; if anxiety is not effectively regulated, it may become further associated with depression, potentially affecting physical and mental health. Nevertheless, this inference awaits confirmation through longitudinal research.

However, in peer groups, the chain mediation path is not significant, but anxiety as an independent mediator plays a significant role and is a part of the mediator. At the same time, family support still retains a significant direct effect on somatic symptoms, which also verifies H3. In the serial mediation model, the depression-only mediation path showed a positive sign in the peer group but a negative sign in the general group. However, the effect sizes in both groups were close to zero, with confidence intervals containing or approaching zero, indicating that the effects were statistically unreliable. The sign reversal primarily reflects the absence of a meaningful effect rather than a substantive difference in mediation patterns.

First, peer supporters who have been systematically trained have mastered strategies for identifying anxiety signals, relaxation techniques, and cognitive restructuring, and are also equipped to recognize depressive tendencies and provide emotional support. These acquired skills may enable them to respond more effectively to early emotional distress. Second, in the general student group, anxiety showed a strong association with depression; in the peer counselor group, however, the serial indirect effect via anxiety and depression was not significant. This pattern suggests that peer counselors may differ from general students in how anxiety and depression are linked in relation to family support and somatic symptoms, though the specific mechanisms underlying this difference require further investigation. Studies on eating behavior have similarly found that anxiety and depression play a serial mediating role between family support and eating outcomes (Xue et al., 2026), suggesting that this emotional cascade pattern may be relevant across various physical and mental health outcomes. In addition, the retention of a significant direct effect of family support in the peer group may be related to the psychological resources accumulated through helping practices, which could facilitate a more direct association between family support and psychological adaptation, consistent with the gain spiral mechanism (Hakanen et al., 2008).

To sum up, the ordinary student group relied on the anxiety–depression serial mediation pathway, whereas the peer counselor group exhibited an independent mediation pathway through anxiety along with a significant direct effect. This pattern suggests potential directions for mental health support strategies in higher education: for ordinary students, early identification of anxiety and comprehensive management of the emotional chain may be particularly relevant; for peer counselors, reinforcing their existing psychological skills and resources could be considered to maintain their current advantages.

## 5. Conclusions

Using a large sample, this study compared differences between the peer group and the general student group in the pathways through which family support is associated with somatic symptoms. The findings indicated that the peer group reported higher levels of perceived family support and fewer depressive, anxiety, and somatic symptoms. Depression partially mediated the association between family support and somatic symptoms in both groups, with no significant between-group difference in the indirect effect. However, the direct effect was relatively larger in the peer group, suggesting a more prominent direct association between family support and somatic symptoms in this group. In the serial mediation model, peer counselors showed a significant independent mediation pathway through anxiety alongside a significant direct effect, whereas general students exhibited a significant serial mediation pathway through anxiety and depression.

This study innovatively revealed that the peer counselors’ advantages in mental health indicators may be related to their systematic psychological training and helping practice experiences. Specifically, peer counselors may benefit through the independent pathway of anxiety alleviation, while also showing a weakened serial mediation via anxiety and depression. In contrast, general students rely more heavily on the complete anxiety–depression serial pathway. These findings extend the group-level applicability of social support theory and provide preliminary implications for mental health support strategies in higher education: strategies for general students could focus on depression management and comprehensive emotional support, whereas those for peer counselors might further strengthen their existing psychological skills and resources. However, these implications are derived from correlational findings and await validation in future intervention studies.

The primary limitations of this study are as follows. First, the cross-sectional design precludes causal inferences regarding the observed associations. Second, the assignment of participants to the peer group was non-randomized, which may introduce self-selection bias and unmeasured confounding factors that threaten the internal validity of the between-group comparisons. Third, all data were derived from self-report measures, which may be subject to common method bias. Fourth, measurement invariance across groups was not formally tested. Finally, due to the absence of group-level identifiers (e.g., class or department), clustering effects could not be statistically evaluated; nevertheless, the single-institution sampling and the relatively dispersed distribution across grades and majors may help mitigate this concern.

Future studies may adopt longitudinal follow-up designs, expand cross-regional samples, refine subgroup analyses within peer groups, and simultaneously examine the interaction between negative and positive psychological variables to further validate the dynamic processes of peer empowerment.

## Figures and Tables

**Table 1:** Basic Information of Participants.

	Peer Counselors		General Students	
	Male	Female	Male	Female
Freshman year	557	301	1127	499
Sophomore year	620	255	1171	411
Junior year	318	203	837	384
Senior year	183	139	738	279

**Table 2:** Comparison of Differences in Family Support, Anxiety, Depression, and Somatic Symptoms Between Peer Counselors and General Students.

Variable	Peer Counselors (*n* = 2576)	General Students (*n* = 5446)	t	95% CI	Cohen’s d
	M ± SD	M ± SD			
**Family Support**	19.97 ± 4.95	18.90 ± 5.36	8.56 ***	[0.83, 1.32]	0.21
**Anxiety**	5.56 ± 6.62	6.49 ± 7.14	−5.56 ***	[−0.63, −0.30]	−0.13
**Depression**	5.17 ± 6.86	6.51 ± 7.57	−7.61 ***	[−0.84, −0.50]	−0.18
**Somatic Symptoms**	1.61 ± 2.33	1.75 ± 2.46	−2.34 **	[−0.25, −0.02]	−0.06

Note: ** *p* < 0.01, *** *p* < 0.001.

**Table 3:** Correlation Coefficients of Variables in Peer Counselors and General Students.

	Peer Counselors	Family Support	Anxiety	Depression	Somatic Symptoms
General Students					
Family Support			−0.298 ***	−0.325 ***	−0.190 ***
Anxiety		−0.260 ***		0.866 ***	0.518 ***
Depression		−0.295 ***	0.862 ***		0.426 ***
Somatic Symptoms		−0.149 ***	0.491 ***	0.437 ***	

Note: *** *p* < 0.001. Correlations for general students are presented below the diagonal, and those for peer counselors above the diagonal.

**Table 4:** Results of the Test of the Mediating Effect of Depression Between Family Support and Somatic Symptoms.

Path	Peer Counselors				General Students			
	Effect Value	BootSE	95% CI	Ratio	Effect Value	BootSE	95% CI	Ratio
Direct Effect	−0.062 **	0.021	[−0.104, −0.021]	31.6%	−0.027 *	0.013	[−0.052, −0.002]	17.4%
Indirect Effect	−0.134 ***	0.013	[−0.161, −0.109]	68.4%	−0.128 ***	0.008	[−0.145, −0.112]	82.6%
Total Effect	−0.196 ***	0.023	[−0.242, −0.151]	100.0%	−0.155 ***	0.015	[−0.184, −0.126]	100.0%

Note: * *p* < 0.05, ** *p* < 0.01, *** *p* < 0.001.

**Table 5:** Results of the Chain-Mediation Regression Analyses.

Criterion	Predictor	Peer Counselors					General Students				
		β	SE	t	R^2^	F	β	SE	t	R^2^	F
**Depression**	Gender	−0.017	0.018	−0.936	0.093	87.94 ***	0.026	0.012	2.108 *	0.070	136.49 ***
	Grade	0.058	0.019	2.976 **			0.048	0.013	3.623 ***		
	Family Support	−0.300	0.023	−12.889 ***			−0.259	0.015	−17.148 ***		
**Anxiety**	Gender	0.054	0.010	5.447 ***	0.758	2013.81 ***	0.030	0.007	4.377 ***	0.749	4076.43 ***
	Grade	−0.010	0.010	−1.019			−0.015	0.007	−2.165 *		
	Family Support	−0.071	0.011	−6.544 ***			−0.075	0.008	−9.943 ***		
	Depression	0.845	0.015	56.283 ***			0.842	0.008	100.441 ***		
**Somatic Symptoms**	Gender	−0.104	0.017	−6.105 ***	0.283	203.13 ***	−0.105	0.012	−8.863 ***	0.254	371.37 ***
	Grade	0.025	0.017	1.510			0.026	0.012	2.202 *		
	Family Support	−0.051	0.020	−2.475 *			−0.024	0.012	−1.926		
	Depression	0.572	0.046	12.457 ***			0.434	0.031	14.132 ***		
	Anxiety	−0.081	0.043	−1.880			0.062	0.029	2.127 *		

Note: * *p* < 0.05, ** *p* < 0.01, *** *p* < 0.001.

**Table 6:** Test of the serial mediation effect of anxiety and depression between family support and somatic symptoms.

Path		Peer Counselors			General Students		
		Effect Value	BootSE	95% CI	Effect Value	BootSE	95% CI
**Direct Effect**	FS → SS	−0.051 *	0.020	[−0.093, −0.012]	−0.024	0.012	[−0.048, 0.000]
**Indirect Effect**	FS → A → SS	−0.172 ***	0.018	[−0.211, −0.139]	−0.112 ***	0.010	[−0.133, −0.094]
	FS → D → SS	0.006	0.003	[−0.0004, 0.013]	−0.005 *	0.002	[−0.009, −0.0003]
	FS → A → D → SS	0.021	0.011	[−0.001, 0.043]	−0.013 *	0.006	[−0.026, −0.001]
**Total Indirect Effect**		−0.145 *	0.013	[−0.172, −0.120]	−0.130 *	0.011	[−0.147, −0.115]
**Total Effect**		−0.196 ***	0.023	[−0.241, −0.151]	−0.154 ***	0.015	[−0.184, −0.126]

Note: * *p* < 0.05, *** *p* < 0.001.

**Table 7:** Results of the test of the mediating effect of anxiety between family support and somatic symptoms among peer counselors.

Path	Peer Counselors			
	Effect Value	BootSE	95% CI	Ratio
Direct Effect	−0.045 *	0.020	[−0.086, −0.006]	23.0%
Indirect Effect	−0.151 ***	0.015	[−0181, −0.125]	77.0%
Total Effect	−0.196 ***	0.023	[−0.241, −0.151]	100.0%

Note: * *p* < 0.05, *** *p* < 0.001.

**Table 8:** Tests of between-group differences in the effect sizes of each path in the chain mediation model.

Path		Standardized Difference Estimate (Peer Counselors—General Students)	BootSE	*p*	95% CI
**Indirect Effect**	FS → A → SS	−0.059 **	0.021	0.005	[−0.102, −0.020]
	FS → D → SS	0.010 **	0.004	0.008	[0.003, 0.019]
	FS → A → D → SS	0.034 **	0.013	0.008	[0.010, 0.060]
**Direct Effect**	FS→SS	−0.027	0.024	0.256	[−0.075, 0.018]
**Total Effect**		−0.042	0.027	0.123	[−0.095, 0.011]

Note: ** *p* < 0.01.

## Data Availability

The data that support the findings of this study are available from the corresponding author upon reasonable request.

## Supplementary Materials

The following supporting information can be downloaded at: https://www.mdpi.com/article/10.3390/bs16091465/s1, Mplus command syntax S1: Multi-group Mediation Analysis of Simple Mediation; Mplus command syntax S2: Multi-group Mediation Analysis of Serial Mediation.
