# Supplementary material for: Family Support and Somatic Symptoms: The Serial Mediating Roles of Anxiety and Depression—A Multi-Group Comparison Between Peer Counselors and General Students
Source: Behav Sci (Basel). 2026 Aug 24;16(9):1465. doi: 10.3390/bs16091465 (PMC13603766; doi:10.3390/bs16091465)
Supplement: Supplementary file 1 [file behavsci-16-01465-s001.zip › behavsci-4466960-supplementary.pdf]

Supplementary Materials S1

Mplus command syntax S1: Multi-group Mediation Analysis of Simple Mediation

```
TITLE: Multi-group mediation analysis: includes control variables and the total
effect ;

DATA:
   FILE = analysis.dat;

VARIABLE:
   NAMES = gender grade group anxiety M X Y;
   GROUPING = group (0 = general 1 = peer);
   USEVARIABLES = gender grade X M Y;
   MISSING = .;

ANALYSIS:
   ESTIMATOR = ML;
   BOOTSTRAP = 5000;

MODEL:
   M ON X gender grade;
   Y ON X M gender grade;

MODEL PEER:
   M ON X (a_peer);
   Y ON M (b_peer);
   Y ON X (c_peer);

MODEL GENERAL:
   M ON X (a_general);
   Y ON M (b_general);
   Y ON X (c_general);

MODEL CONSTRAINT:
   NEW (sd_x_ge sd_y_ge sd_x_pe sd_y_pe
         ind_peer ind_general diff_ind
         dir_peer dir_general diff_dir
         total_peer total_general diff_total
         ind_peer_std ind_general_std diff_ind_std
         dir_peer_std dir_general_std diff_dir_std
```

```
          total_peer_std total_general_std diff_total_std);
   sd_x_ge = 5.361;
   sd_y_ge = 2.456;
   sd_x_pe = 4.953;
   sd_y_pe = 2.327;

   ! ---- Non-standardized effect ----
   ind_peer      = a_peer * b_peer;
   ind_general = a_general * b_general;
   diff_ind      = ind_peer - ind_general;

   dir_peer      = c_peer;
   dir_general = c_general;
   diff_dir      = dir_peer - dir_general;

   total_peer      = dir_peer + ind_peer;
   total_general = dir_general + ind_general;
   diff_total      = total_peer - total_general;

   ! ---- Standardization Effect ----
   ind_peer_std      = ind_peer      * (sd_x_pe / sd_y_pe);
   ind_general_std = ind_general * (sd_x_ge / sd_y_ge);
   diff_ind_std      = ind_peer_std - ind_general_std;

   dir_peer_std      = dir_peer      * (sd_x_pe / sd_y_pe);
   dir_general_std = dir_general * (sd_x_ge / sd_y_ge);
   diff_dir_std      = dir_peer_std - dir_general_std;

      total_peer_std      = ind_peer_std + dir_peer_std;
      total_general_std = ind_general_std + dir_general_std;
      diff_total_std      = total_peer_std - total_general_std;


OUTPUT:
   SAMPSTAT;
   STANDARDIZED;
   CINTERVAL(BCBOOTSTRAP);
```

Mplus command syntax S2: Multi-group Mediation Analysis of Serial Mediation

```
TITLE: Multiple chain-type intermediates X-M-M2-Y, with standardized effects;

DATA:
   FILE = analysis.dat;

VARIABLE:
   NAMES = gender grade group M M2 X Y;
   GROUPING = group (0 = general 1 = peer);
   USEVARIABLES = gender grade X M M2 Y;
   MISSING = .;

ANALYSIS:
   ESTIMATOR = ML;
   BOOTSTRAP = 5000;

MODEL:
   M ON X gender grade;
   M2 ON X M gender grade;
   Y ON X M M2 gender grade;

MODEL PEER:
   M ON X (a1_p);
   M2 ON X (a2_p);
   M2 ON M (d_p);
   Y ON M (b1_p);
   Y ON M2 (b2_p);
   Y ON X (c_p);

MODEL GENERAL:
   M ON X (a1_t);
   M2 ON X (a2_t);
   M2 ON M (d_t);
   Y ON M (b1_t);
   Y ON M2 (b2_t);
   Y ON X (c_t);

MODEL CONSTRAINT:
   NEW (sd_x_ge sd_y_ge sd_x_pe sd_y_pe
          ind1_p ind2_p chain_p dir_p total_p
```

```
        ! General group effect (non-standardized)
        ind1_t ind2_t chain_t dir_t total_t
        ! Standardization effect
        ind1_p_std ind2_p_std chain_p_std dir_p_std total_p_std
        ind1_t_std ind2_t_std chain_t_std dir_t_std total_t_std
        ! Standardization Differences
        diff_ind1_std diff_ind2_std diff_chain_std diff_dir_std diff_total_std
        ! Non-standardized difference


sd_x_ge = 5.361;
sd_y_ge = 2.456;
sd_x_pe = 4.953;
sd_y_pe = 2.327;

! ========== Non-standardized effect    ==========
! peer group
ind1_p = a1_p * b1_p;
ind2_p = a2_p * b2_p;
chain_p = a1_p * d_p * b2_p;
dir_p = c_p;
total_p = dir_p + ind1_p + ind2_p + chain_p;

! general group
ind1_t = a1_t * b1_t;
ind2_t = a2_t * b2_t;
chain_t = a1_t * d_t * b2_t;
dir_t = c_t;
total_t = dir_t + ind1_t + ind2_t + chain_t;

! ========== Standardization effect ==========
! peer group
ind1_p_std     = ind1_p      * (sd_x_pe / sd_y_pe);
ind2_p_std     = ind2_p      * (sd_x_pe / sd_y_pe);
chain_p_std    = chain_p     * (sd_x_pe / sd_y_pe);
dir_p_std      = dir_p       * (sd_x_pe / sd_y_pe);
   total_p_std    = ind1_p_std + ind2_p_std + chain_p_std + dir_p_std;

! general group
ind1_t_std     = ind1_t      * (sd_x_ge/ sd_y_ge);
ind2_t_std     = ind2_t      * (sd_x_ge / sd_y_ge);
chain_t_std    = chain_t     * (sd_x_ge / sd_y_ge);
dir_t_std      = dir_t       * (sd_x_ge / sd_y_ge);
total_t_std    = ind1_t_std + ind2_t_std + chain_t_std + dir_t_std;
```

```
  diff_ind1_std      = ind1_p_std      - ind1_t_std;
  diff_ind2_std      = ind2_p_std      - ind2_t_std;
  diff_chain_std    = chain_p_std    - chain_t_std;
  diff_dir_std       = dir_p_std       - dir_t_std;
  diff_total_std    = total_p_std    - total_t_std;

OUTPUT:
  SAMPSTAT;
  STANDARDIZED;
  CINTERVAL(BCBOOTSTRAP);
```
